# Supplementary figures and images for: Gap junctions mediate discrete regulatory steps during fly spermatogenesis
Source: PLoS Genet. 2022 Sep 29;18(9):e1010417. doi: 10.1371/journal.pgen.1010417 (PMC9578636; doi:10.1371/journal.pgen.1010417)

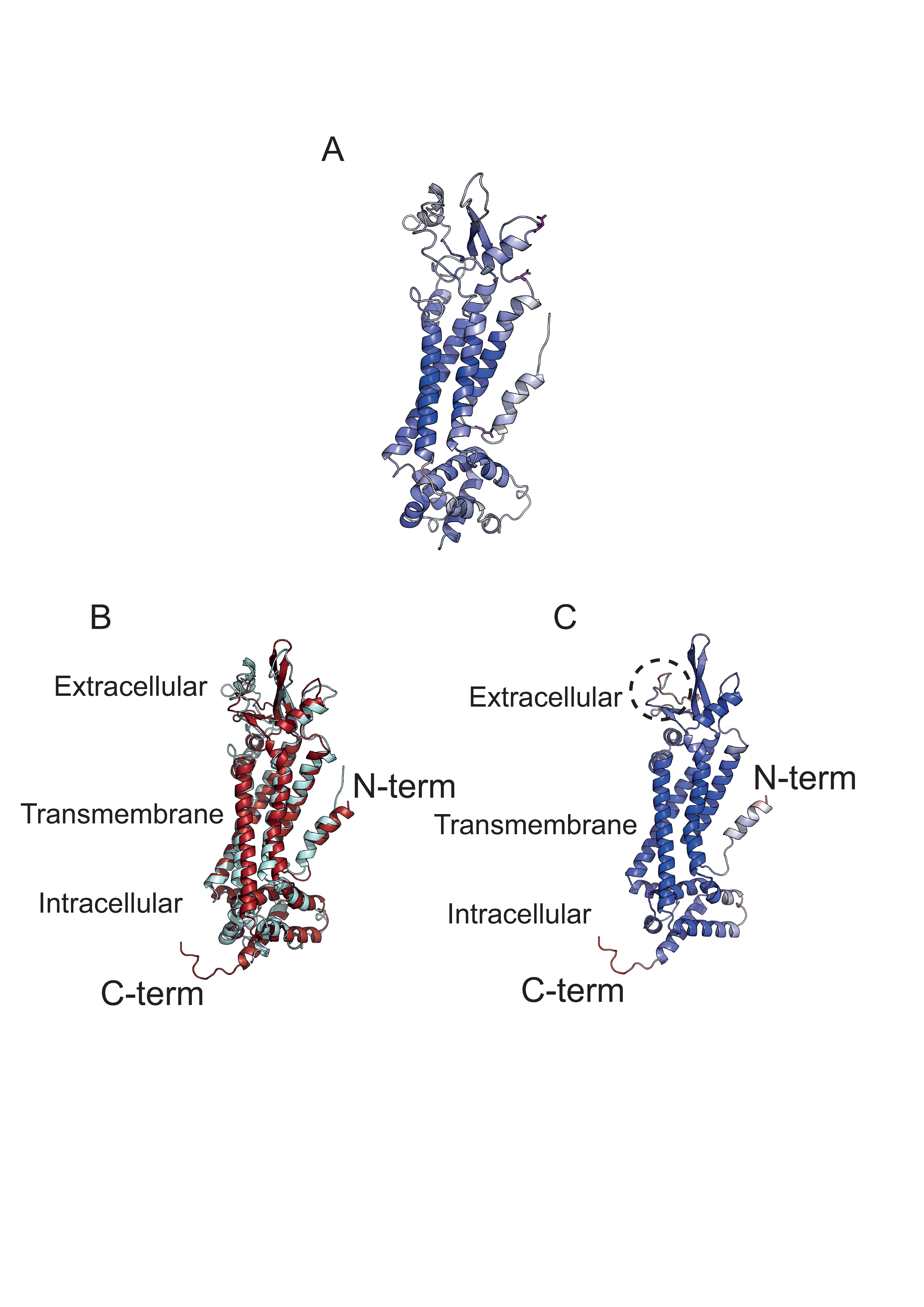

Supplement: S1 Fig — a) Model of a single subunit of D.melanogaster Inx4, color coded according to reliability on a scale from dark red (0% Swiss Model score) to dark blue (70% or higher). This shows that the transmembrane region is the most reliable. Of note, a very similar model is obtained through Alphafold (B, C), adding further confidence to the overall fold and general location of the three Asp residues investigated in this study. The positions of Asp21, Asp50, and Asp59 are indicated via purple sticks. (B) Superposition of models for D.melanogaster Inx4 derived from homology modeling (cyan) and through Alphafold2 (red), showing excellent agreement in the fold, especially for the transmembrane region. (C) model for one subunit of Inx4 obtained through Alphafold2, color coded according to reliability score (from 40% in red, to 100% in dark blue). The areas with lowest reliability include the N-terminus and C-terminus, and a small extracellular loop (circled). As expected, these areas also show the largest divergence between the two models. (TIF) [file pgen.1010417.s001.tif]

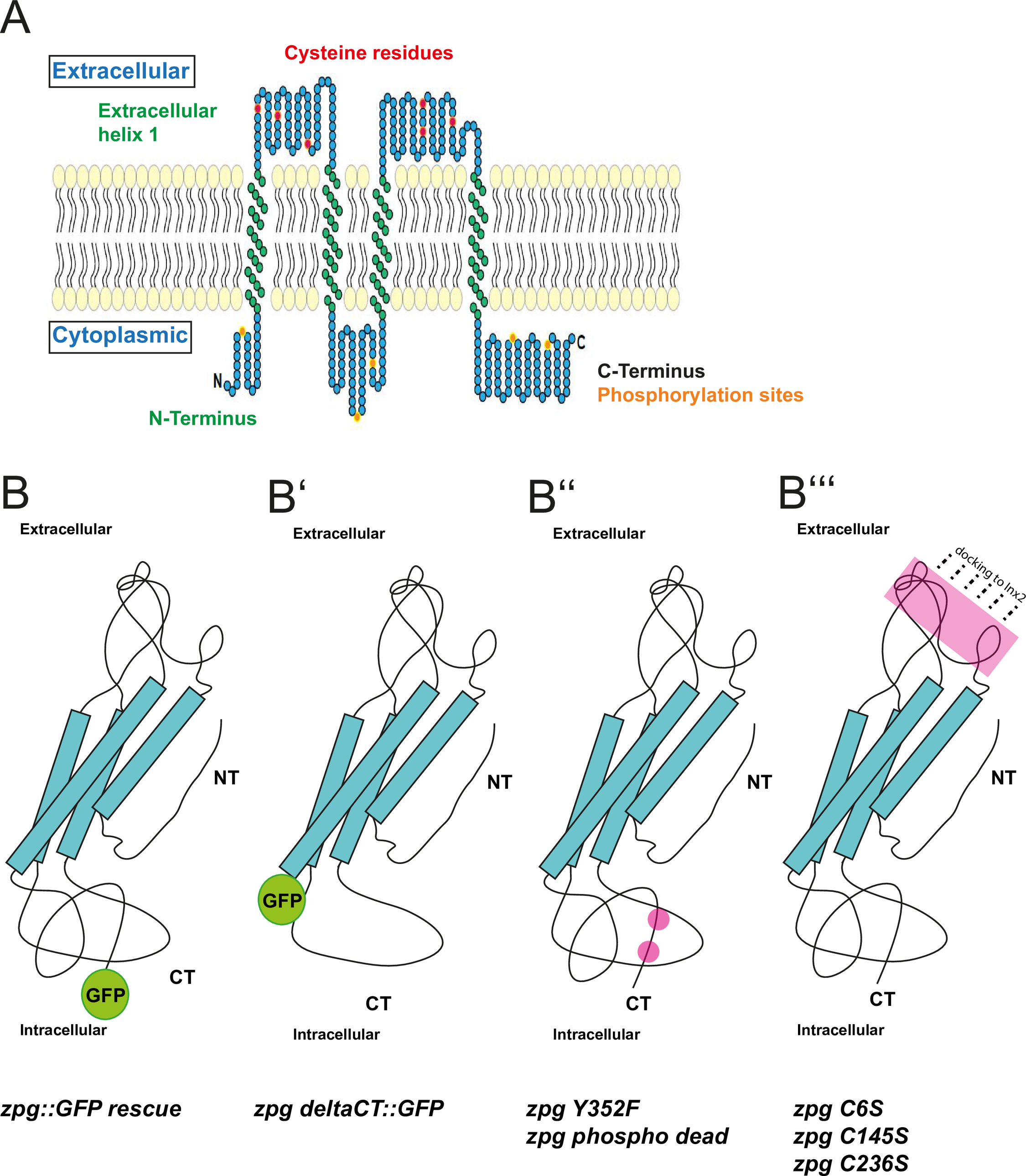

Supplement: S2 Fig — (A) 2D plot of the topology of Zpg marking residues and domains of interest for our structure-function analysis. The Zpg gap junction is a 4-pass transmembrane protein with intracellular N- and C-termini. While the N-terminus and the first extracellular helix are predicted to face inside the channel pore where they likely regulate channel gating, the C-terminus likely has a channel-independent function and contains two phosphorylation sites for potential phospho signaling. It is known from other innexins that cysteine residues in the extracellular region mediate the coupling of two hemichannels in adjacent cells. Here, Zpg in the germ cell membrane couples to Inx2 in the soma cell membrane. (B-B”’) Simplified schematics of the Zpg protein showing the site of GFP-tag insertion for the wildtype (zpg::GFP GR rescue flies, B), and C-terminal deletion mutant (zpg::GFP deltaCT; B’). Also shown are sites of residues altered in the phosphorylation mutants (zpg Y352F and zpg Y352F/S356A; B”) and three different mutations of extracellular cysteines (zpg C6S, zpg C145S, zpg C236S; B”’). (TIF) [file pgen.1010417.s002.tif]

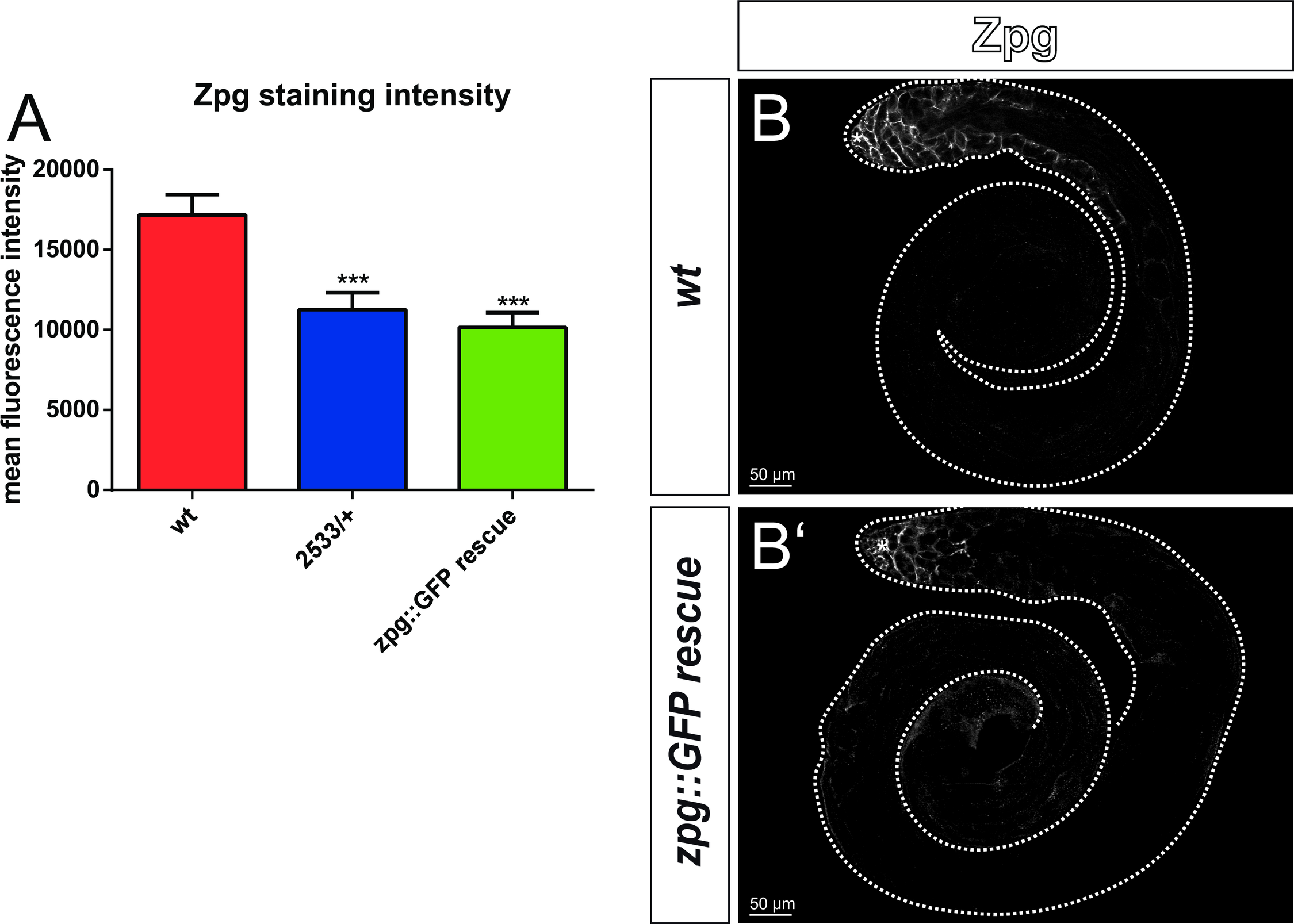

Supplement: S3 Fig — (A) Fluorescence intensity quantification (n = 12 images measured per genotype; mean fluorescence intensity in arbitrary units) of Zpg antibody staining in testes of wildtype control, flies heterozygous for the zpg2533 null allele (zpg2533/+) and zpg mutants rescued with one copy of the genomic rescue construct (zpg::GFP GR). Similar levels of expression are observed in testes of zpg mutants rescued with zpg::GFP GR and zpg2533 heterozygotes. (B-B’) Representative images showing expression levels for wildtype (B) and zpg mutants rescued with zpg::GFP GR (B’). Hubs are marked by asterisks. Scale bars represent 50 μm. p-values are indicated by asterisks with *p<0.05, **p<0.01, ***p<0.001. (TIF) [file pgen.1010417.s003.tif]

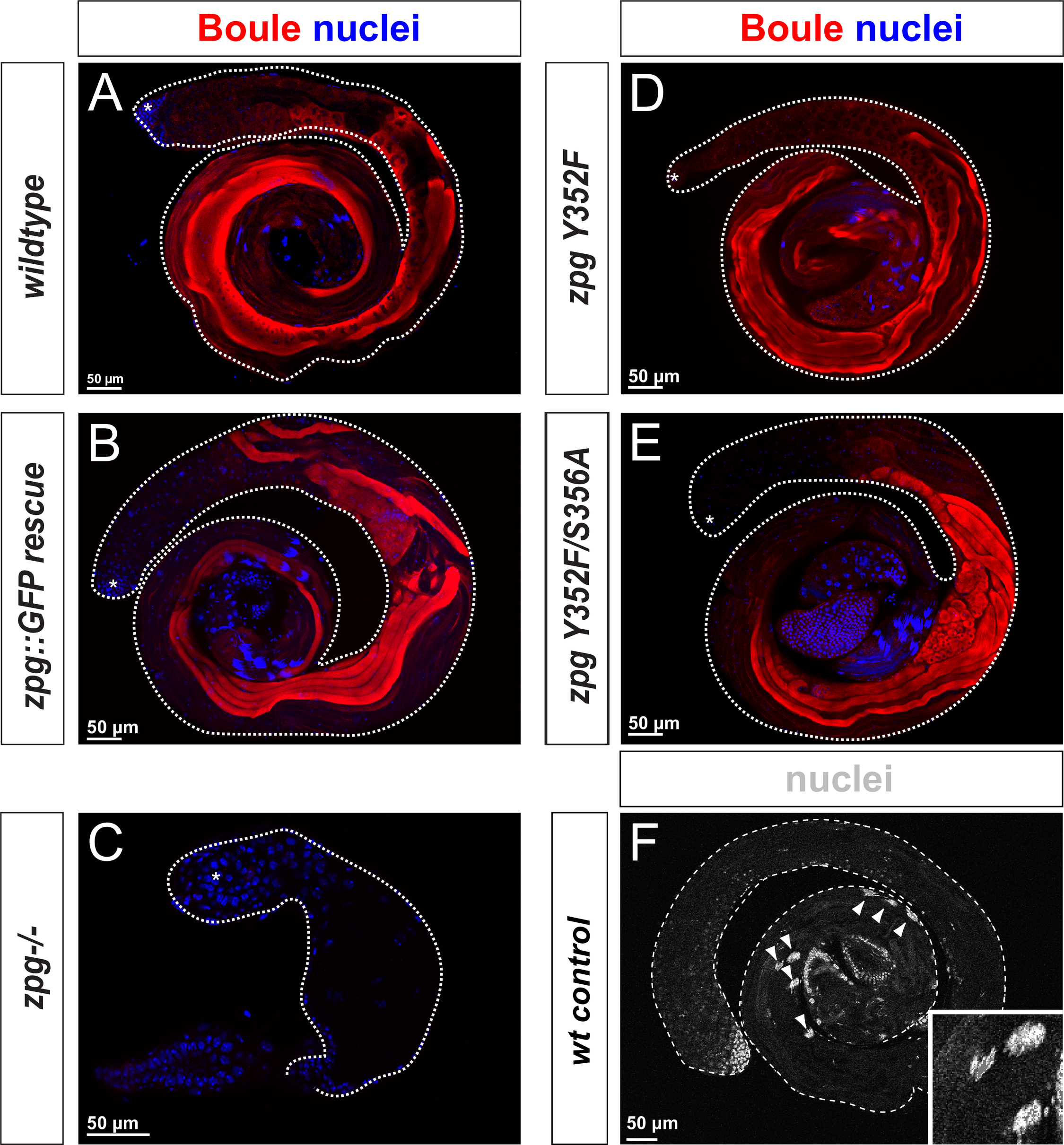

Supplement: S4 Fig — (A-D) Testes stained for the meiotic germ cell marker Boule (red) and nuclei (blue). In testes of wildtype flies (A) or from zpg mutants rescued with zpg::GFP GR (B), which show a complete rescue of the zpg null phenotype, Boule signal is found in late germ cell cysts as well as in long and parallel spermatid bundles. In zpg null mutants (C), meiotic stages are not reached hence no Boule signal is detected. Testes of both phosphorylation site mutants (zpg Y352F in D, zpg Y352F/S356A in E) show strong Boule signal and the parallel organization of spermatid bundles. Hubs are marked by asterisks. (F) Testes stained for nuclear marker (white), arrows indicate elongated spermatid bundles. Scale bars represent 50 μm. (TIF) [file pgen.1010417.s004.tif]

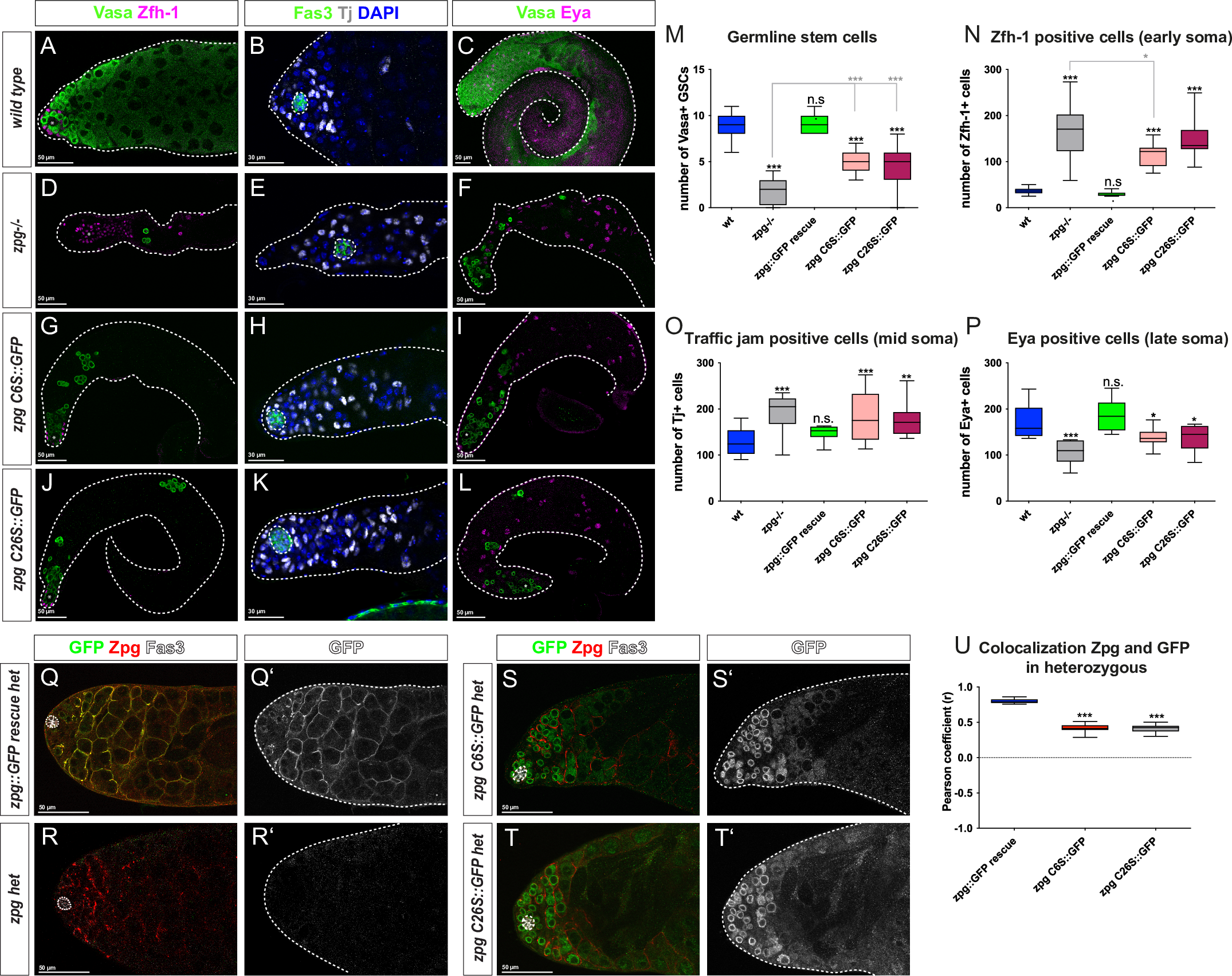

Supplement: S5 Fig — (A-L) Immunostaining for germ and somatic cells in wild type (A-C), zpg null mutant (D-F), zpg null mutant rescued with zpg::GFP C6S (G-I) and zpg null mutant rescued with zpg::GFP C26S (J-L). Both C6S::GFP and C26S::GFP rescue exhibit a phenotype that is indistinguishable from the zpg null mutant with a decreased number of germ cells (Vasa+) and late somatic cells (Eya+) as well as an increased number of early-mid somatic cells (Zfh-1+ and Tj+). The immunostainings, as well as the quantification of (M) germline stem cells (GSCs), (N) Zfh-1-positive cells, (O) Tj-positive cells and (P) Eya-positive cells reveals a strong, nearly null-mutant like phenotypes in zpg::GFP C6S and zpg::GFP C26S rescued testes, indicating a strong loss of function. This is consistent with the absence of the mutated Zpg::GFP C6S and Zpg::GFP C26S proteins from the plasma membrane (S-U). (Q-T) Colocalization of endogenous, unmutated Zpg and GFP-tagged, mutated Zpg in flies with one copy of endogenous Zpg. zpg GFP::GR and zpg heterozygous controls are depicted in B-B’ and C-C’, respectively. In testes expressing the zpg::GFP C6S (S-S’) and zpg::GFP C26S (T-T’) mutant constructs, the GFP-tagged mutated Zpg accumulates intracellularly, while endogenous Zpg mainly localizes to the plasma membrane. This results in low Pearson colocalization coefficients (U) upon quantification of the colocalization of endogenous and mutated Zpg testes of flies with the zpg::GFP C6S and zpg::GFP C26S mutant transgenes compared to the zpg::GFP GR control. (TIF) [file pgen.1010417.s005.tif]

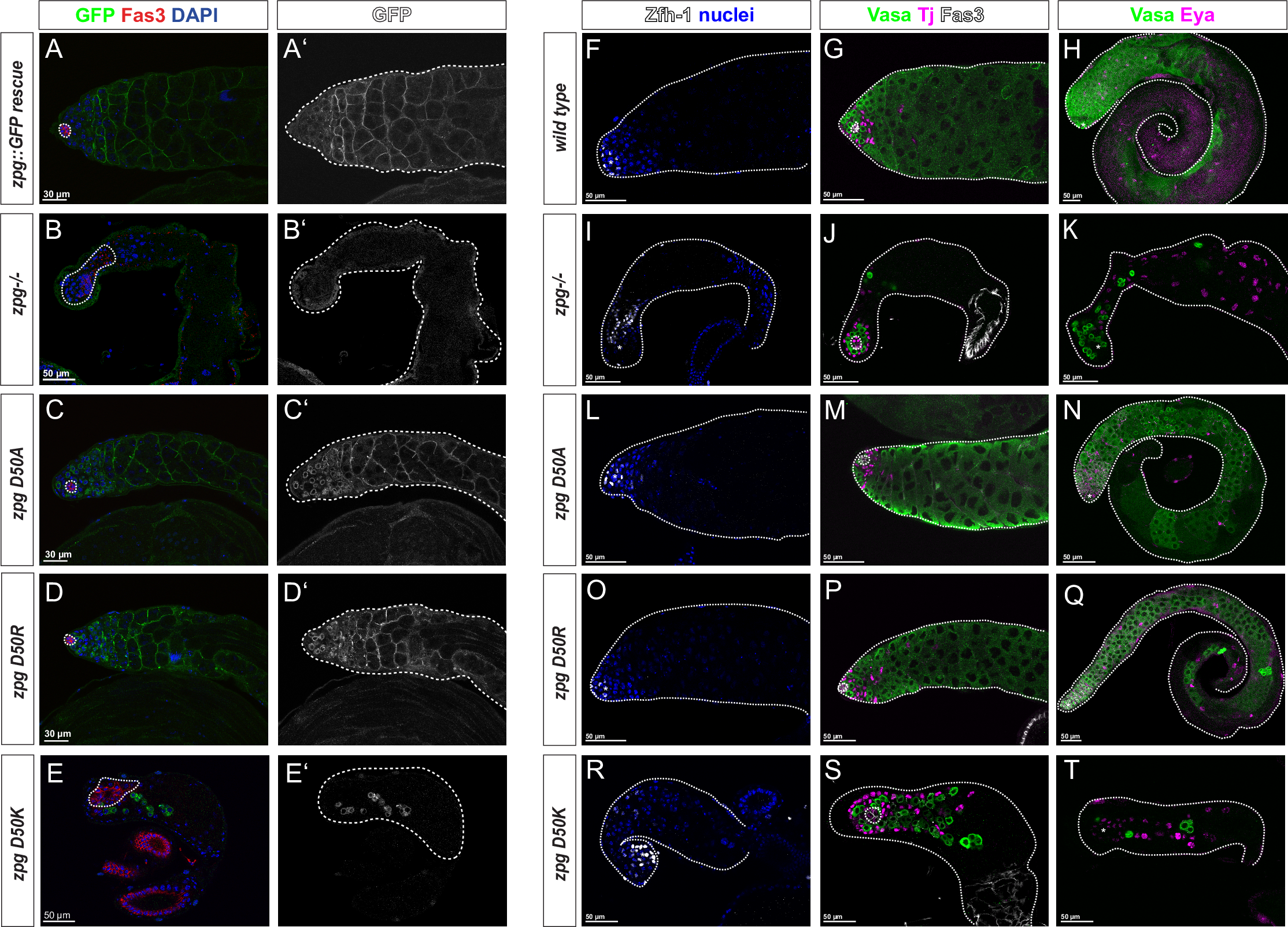

Supplement: S6 Fig — (A-E) The subcellular localization of mutant Zpg proteins is revealed by staining for GFP (green) as all rescue transgenes contain a GFP tag at the C-terminus of Zpg, Fas3 (red) is used to mark the hub and nuclei are stained with DAPI (blue). GFP single channel is depicted in in white (A’-E’). zpg::GFP GR rescue control (A-A’) shows GFP localization at the membrane. Zpg null mutants (B-B’) do not express GFP. In testes of zpg mutants rescued with the zpg D50A (C-C’) or zpg D50R (D-D’) rescue constructs, which show mild germ cell differentiation defects, the GFP signal is strongly enriched at the plasma membrane, indicating normal localization of the mutant GJ proteins. The low number of germ cells in testes of zpg mutants rescued with the zpg D50K mutant rescue construct (E-E’) makes it hard to determine the localization of the GFP-tagged mutant Zpg protein. Therefore, localization of this mutant was analyzed in a heterozygous background, containing one copy of the endogenous zpg, this data is shown in Fig 5. (F-T) Analysis of germ cell and somatic cell markers in the N-terminal mutant rescue constructs. Staining for Vasa (mitotic germ cells, left and right panel; green) and the somatic markers Zfh-1 (early soma, left panel; grey), Tj (early-mid soma, middle panel; magenta) and Eya (late soma, right panel; magenta). Hubs are marked with Fas3 in grey, nuclei are labelled in blue. Wild type control depicted in F-H, zpg null mutant in I-K. The number of early somatic cells (Zfh-1+, Tj+) in the testes of zpg mutants rescued with either zpg D50A (L, M), zpg D50R (O, P) or zpg D50K rescue constructs (R, S). All analyzed mutants have a lower number of Eya+ cells than wt (right panel). Associated quantifications are shown in Fig 5. Hubs are marked with dashes. Scale bars represent 50 μm. (TIF) [file pgen.1010417.s006.tif]

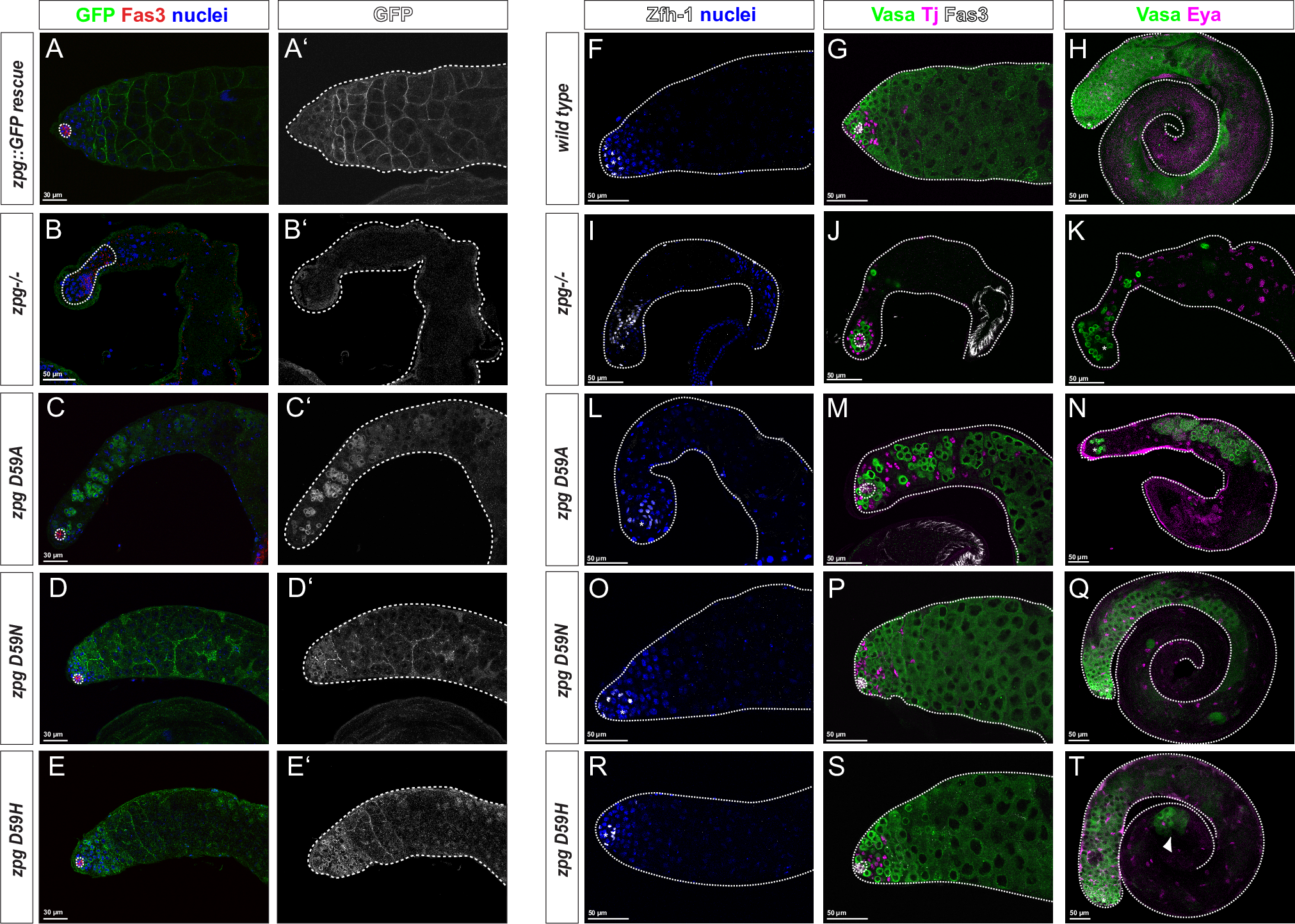

Supplement: S7 Fig — (A-E) The subcellular localization of mutant Zpg proteins is revealed by staining for GFP (green) as all rescue transgenes contain a GFP tag at the C-terminus of Zpg, Fas3 (red) is used to mark the hub and nuclei are stained with DAPI (blue). GFP single channel is depicted in in white (A’-E’). zpg::GFP GR rescue control (A-A’) shows GFP localization at the membrane. zpg null mutants (B-B’) do not express GFP. The low number of germ cells in testes of zpg mutants rescued with the zpg D59A mutant rescue construct (E-E’) makes it hard to determine the localization of the GFP-tagged mutant Zpg protein. In testes of zpg null mutants rescued with the zpg D59N (D-D’) or the zpg D59H (E-E’) rescue transgenes, the GFP signal is mainly concentrated at the membrane. Therefore, localization of these mutants was analyzed in a heterozygous background, containing one copy of the endogenous zpg, this data is shown in Fig 6. (F-T) Analysis of germ cell and somatic cell markers in the N-terminal mutants. Staining for Vasa (mitotic germ cells, left and right panel; green) and the somatic markers Zfh-1 (early soma, left panel; grey), Tj (early-mid soma, middle panel; magenta) and Eya (late soma, right panel; magenta). Hubs are marked with Fas3 in grey, nuclei are labelled in blue. Wildtype control is depicted in F-H, zpg null mutant in I-K. In testes of zpg null mutants rescued with the D59A rescue construct a strong somatic cell differentiation defect was seen with elevated cell counts for the early somatic markers Zfh-1 (L) and Tj (M), whereas these cell counts appear wildtype for zpg nulls mutants rescues with either the zpg D59N (O, P) or zpg D59H (R, S) rescue construct. All analyzed mutants have a lower number of Eya+ cells compared to wt (right panel). Associated quantifications are shown in Fig 6. Hubs are marked with dashes. Scale bars represent 50 μm. (TIF) [file pgen.1010417.s007.tif]

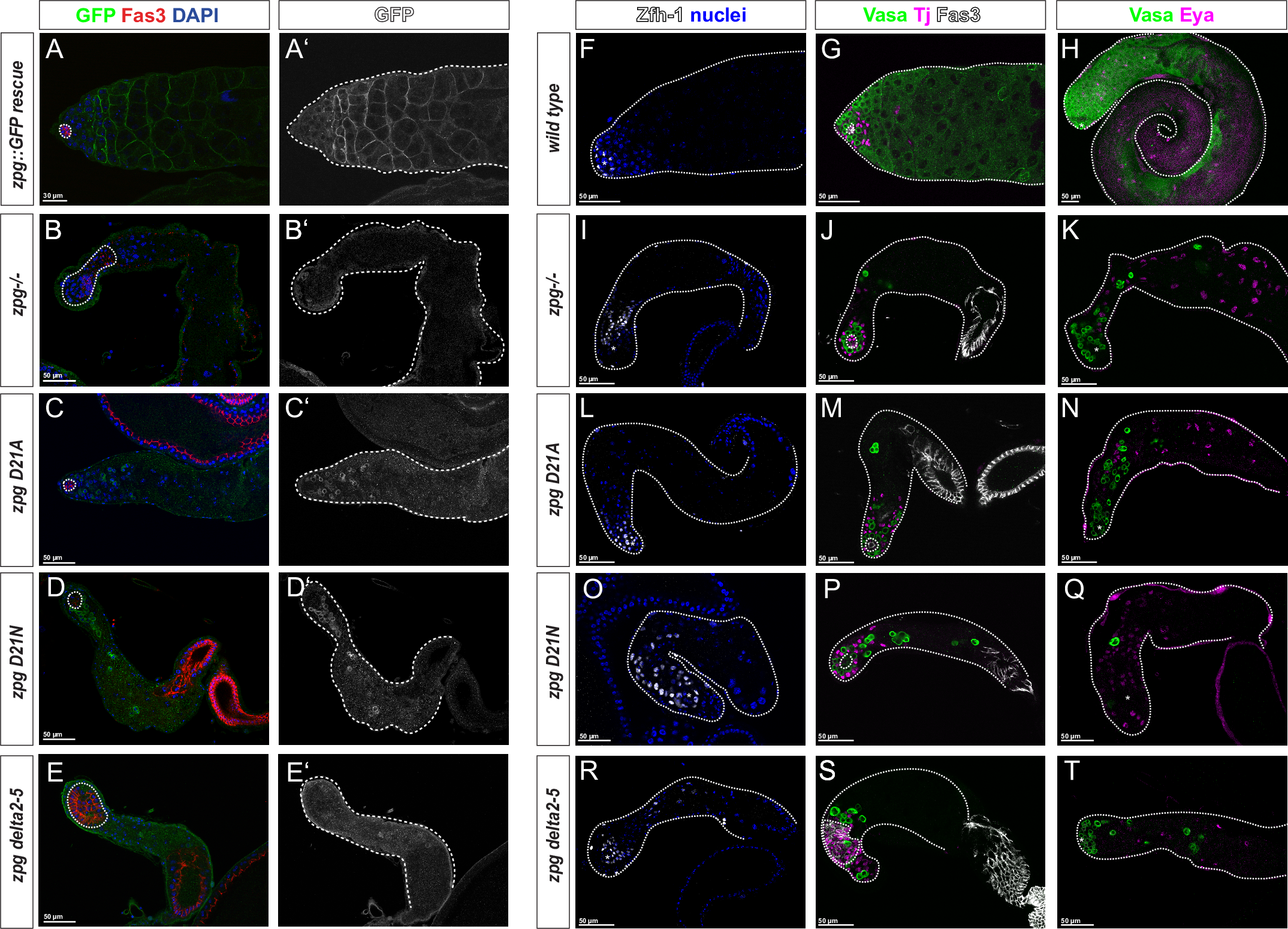

Supplement: S8 Fig — (A-E) The subcellular localization of mutant Zpg proteins is revealed by staining for GFP (green) as all rescue transgenes contain a GFP tag at the C-terminus of Zpg, Fas3 (red) is used to mark the hub and nuclei are stained with DAPI (blue). GFP single channel is depicted in in white (A’-E’). zpg::GFP GR rescue control (A-A’) shows GFP localization at the membrane. zpg null mutants (B-B’) do not express GFP. The low number of germ cells in testes of zpg mutants rescued with either the zpg D21A (C-C’), zpg D21N (D-D’), or zpg delta2-5 rescue constructs (E-E’) makes it hard to determine the localization of the GFP-tagged mutant Zpg protein. Therefore, localization of these mutants was analysed in a heterozygous background, containing one copy of the endogenous zpg, this data is shown in Fig 7. (F-T) Analysis of germ cell and somatic cell markers in the N-terminal mutants. Staining for Vasa (mitotic germ cells, left and right panel; green) and the somatic markers Zfh-1 (early soma, left panel; grey), Tj (early-mid soma, middle panel; magenta) and Eya (late soma, right panel; magenta). Hubs are marked with Fas3 in grey, nuclei are labelled in blue. Wild type control depicted in F-H, zpg null mutant in I-K. Testes of zpg mutants rescues with the zpg D21A (L-N), zpg D21N (O-Q) or zpg delta2-5 (R-T) mutants show a lower number of Vasa-positive germ cells and Eya-positive late somatic cells, while at the same time the number of early somatic cells (Zfh-1- and Tj-positive) is increased. This phenotype, seen in all three N-terminal mutants is indistinguishable from that of the zpg null. Associated quantification are shown in Fig 7. Hubs are marked with dashes. Scale bars represent 50 μm. (TIF) [file pgen.1010417.s008.tif]

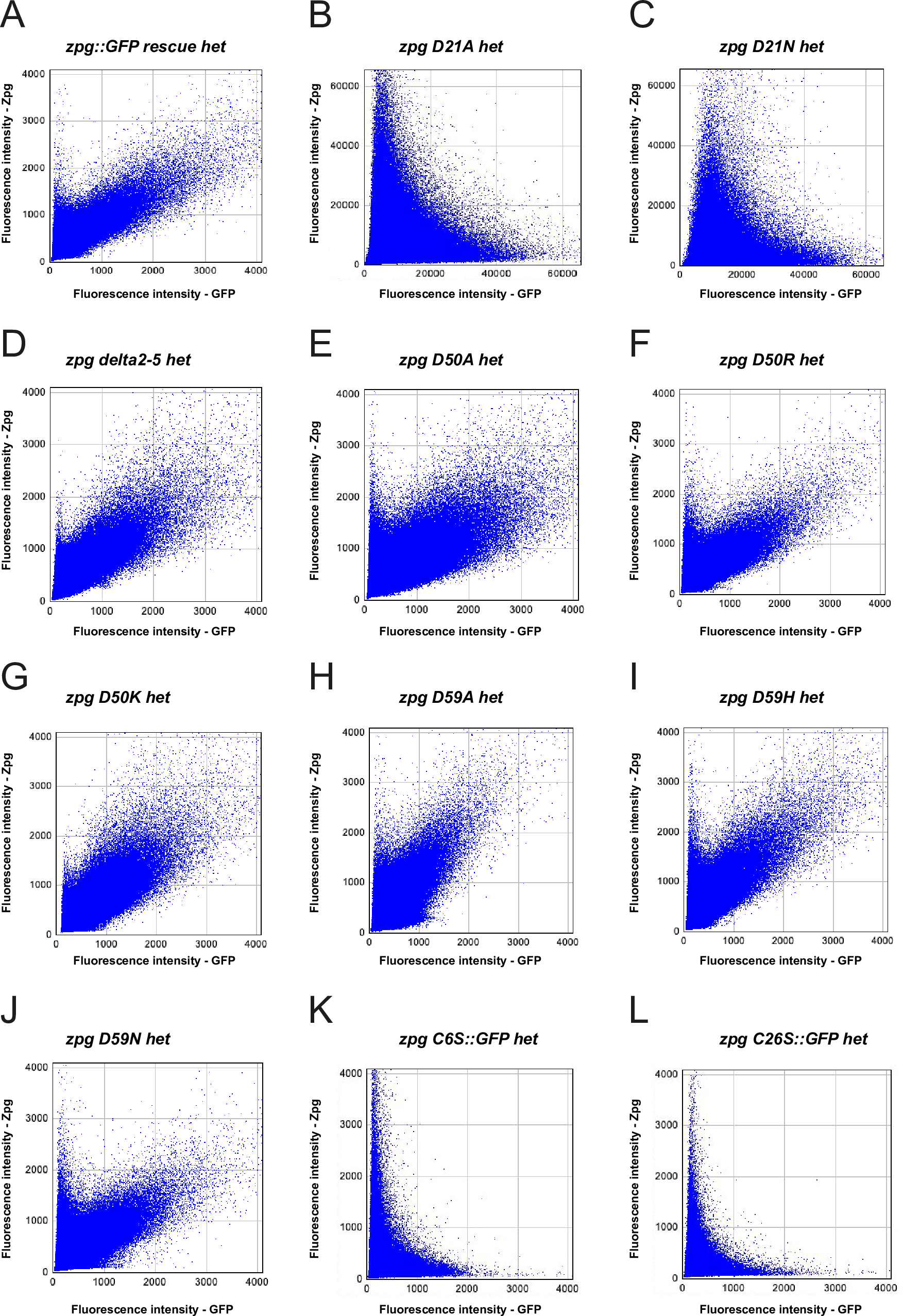

Supplement: S9 Fig — Representative scatter plots generated with the JaCoP plugin in FIJI, one shown per genotype. The signal intensity for endogenous Zpg is plotted against the signal intensity of the GFP-tagged mutated Zpg in flies that have one copy of endogenous and one copy of mutated protein. A linear relationship between endogenous and GFP-tagged signal intensity, like in zpg::GFP GR flies (A) indicates strong colocalization of mutated and endogenous Zpg at the membrane. For zpg D21A (B) and zpg D21N (C) a weak colocalization is detected. Strong colocalization, similar to the control in A, is found in heterozygotes of zpg delta2-5 (D), zpg D50A (E), zpg D50R (F), zpg D50K (G), zpg D59A (H), zpg D59H (I) and zpg D59N (J), whereas colocalization is minimal for zpg C6S::GFP (K) and zpg C26S::GFP (L). (TIF) [file pgen.1010417.s009.tif]
