# Supplementary material for: Gap junctions mediate discrete regulatory steps during fly spermatogenesis
Source: PLoS Genet. 2022 Sep 29;18(9):e1010417. doi: 10.1371/journal.pgen.1010417 (PMC9578636; doi:10.1371/journal.pgen.1010417)
Supplement: Attachment — Submitted filename: Pesch et al response to reviewers.docx [file pgen.1010417.s010.docx]

Dear Jean-Rene,

Enclosed for your consideration please find our response to the reviews of our manuscript entitled “**Gap Junctions Mediate Discrete Regulatory Steps During Fly Spermatogenesis.**”. We were very happy to see that the reviewers found our work to be of great interest and we were grateful for the very helpful and constructive feedback we were provided with. Although they had many positive things to say about the previous version of the manuscript the reviewers raised a number of important points that required improvement to the revised manuscript. We agreed with their assessment and have tried our best to improve the manuscript and in the process addressed all the points they raised.

Below is our point by point response to the reviewers comments, outlining all the ways we improved the manuscript in line with their suggestions.

Best regards,

Guy Tanentzapf

**Point by Point Response:**

**Reviewer 1:**

Pesch et al. reports their study on the role of a gap junction protein (Zpg) in the Drosophila spermatogenesis. The role of gap junction-mediated signaling is well known to regulate soma-germline communication (and cell-cell communication, more broadly) but the molecular details remained underexplored. This manuscript presents a thorough study, which generated many point mutations to a gap junction protein (Zpg). They used available structure information to design point mutations in Zpg protein and examined their ability to rescue zpg mutant (and examined mutant phenotypes when the rescue transgene is not fully functional). The study indicates that different mutations affect different stages of germ cell development, providing novel and important insights into how soma-germline interaction carve the process of germ cell differentiation. Specifically, they generate mutants for C-terminal deletion, phosphorylation mutants, Cysteine mutants that are proposed to interfere with GJ formation or cargo selectivity. The results are quite informative and contribute significantly to the field.

1) The study also has an important implication as to why mammalian Sertoli cells, which encapsulate all stages of germ cells, can regulate different steps of germ cell differentiation. This (why single Sertoli cell can regulate spermatogonia and spermatocytes and spermatid at the same time) has been a question many people vaguely asked without any idea. Discussing their finding in this context would be nice.

**Thanks for bringing this to our attention! In the revised manuscript we added a section to the last paragraph of the discussion to talk about this.**

2) Intro 2nd page, 2nd paragraph is extremely confusing. It starts out by calling Innexins and connexins as ‘gap junction’ proteins (where they state that GJ is the connection between hemichannels between two adjacent cells). Then go on to say that innexin’s ORTHOLOG are pannexins, which only forms hemi-channel. The definition of ortholog is they are functionally equivalent. Thus, it reads as if the authors are committing to saying that innexins function as hemi-channel---but the rest of the intro is written as if innexins and connexins are functional equivalents (=orthologs), and much later in the results, they say it is not well known which is the mode of function for innexins. Combination of possible misuse of the term and not-so-straightforward introduction of information throughout the manuscript makes the manuscript quite confusing.

   **We apologize for being unclear, our use of orthologue in this context was indeed incorrect. We have changed that and tried our best to be more precise in our language. We now explain that while Innexins definitely form gap junctions, it was not known prior to our study whether they ALSO formed hemichannels like their orthologs pannexins. Our work suggests that they do not, and rather work exclusively by forming full gap junctions.**
3) Fig 1B should indicate that inx4 is ZPG

   **We have fixed this issue.**
4) Fig2D: I believe that Zpg-GFP colocalizes with endogenous Zpg, but this figure does not quite prove that point. In this genotype, there are Zpg-GFP transgene and wild type (endogenous) Zpg gene. When anti-Zpg is used, it will recognize Zpg-GFP as well as endogenous Zpg protein. And this Zpg-GFP molecule that is also recognized by the antibody will surely co-localize with GFP. Unless endogenous Zpg localizes very different place (such as nucleus, nuclear envelope, ER), one cannot prove ‘colocalization’ by GFP and antibody staining. I am not saying they are wrong, but the point that Zpg-GFP colocalizing with endogenous Zpg can be made simply by comparing Zpg-GFP localization and anti-Zpg staining of the wt. I am not requesting any experiment, but the authors might want to modify the text. Only after reading to the end of Fig4, it is explained that C-ter GFP tag of Zpg protein interferes the recognition by anti-Zpg antibody. This info should actually be provided earlier here.

**We completely agree that we should have mentioned earlier that the C-terminal GFP tag interferes with the ability of the anti-Zpg antibody to detect the protein. This is the basis of our ability to tell apart the staining that comes from the endogenous Zpg and the Zpg-GFP construct. We apologize for being unclear and we now introduce this quirk of the Zpg antibody and C-terminal GFP tag when we describe figure 2D in the text.**

5) -Fig3E vs. 3E”: I don’t know about the journal style, but generally speaking, the images shouldn’t be given the same letter (E vs. E”) if they are different images.

**The reviewer is correct and this issue has been fixed.**

6) Y352F S356A: I don’t like that the double mutant called ‘phospho-dead’---because this term usually refers to a single site (Y352F is also ‘phospho dead’ of Y352F). Also unless phosphorylation status is checked by mass-spec etc., the double mutant Y352F S356A could have some other phosphorylation. One does not need to name a mutant vaguely (which can be misleading), but just calling ‘Y352F, S356A’ is much more accurate.

**We completely agree with the reviewer and have rephrased such that we no longer use the term “phospho dead” and instead refer to these mutants by their proper name (zpg Y352F/S356A mutants).**

7) Based on the images presented in Fig3C and D, localization of Y352F and Y352F S356A seems to be altered (as opposed to the statement in the manuscript). The mutants’ signal on the membrane seems to be less contiguous (patches and foci more prominent than wt). I agree with their statement about that the spermatogenesis appears normal (at least up until meiosis).

**Thanks for pointing this out, we have altered the description of the localization of the mutants in the testes to mention that Zpg localization in the phospho mutants appears somewhat different than wildtype controls (patchy and less contiguous). As the reviewer points out not only does spermatogenesis appear normal until meiosis in these mutants but they are fully fertile.**

8) Fig4: they generate mutants that are supposed to be defective in forming GJ (meaning, the hemichannels cannot engage in interaction with the hemichannels on the other cell). There are a few confusing issues/problems in this figure. First, if panel A-D is correct, Zpg mutants are not expressed at all (or very weakly expressed). This suggests that, these mutations might just interfere with the stability of the protein (before forming channels), and perhaps their conclusion that Zpg functions by forming channels may not be as strongly supported as stated. The text should be modified accordingly. The lack of Zpg Cys mutant expression is somewhat inconsistent with Zpg-Cys-GFP results shown in Fig4E (clearly the same mutant, being only different by the tag).

**This is an excellent question. We will start by addressing the discrepancy between what we learn about the Cysteine mutant expression from Zpg staining and GFP staining. The Zpg antibody, while decent, is not as sensitive as the GFP antibody, which has exquisite sensitivity. This is why we think these mutants are in fact expressed. It is possible that these mutants could interfere with the protein structure but that is unlikely since the cysteine residues are all surface residues and should not interfere with folding or packing of the protein. Nonetheless we added caveats in the text to insert a note of caution about the interpretation of these experiments.**

9) Fig5-7: it is hard to follow when they talk about the data from these 3 figures, going back and forth.

**We are aware of this challenge, it was not easy to summarize this large amount of complex data. We actually tried multiple ways of discussing this data and ended up on the compromise on display in the current version. We hope that when typeset in PLoS genetics format it will be easier to follow without as much back and forth.**

10) Fig5: panel L and M cannot be the same category of the phenotype (in L, the testis is filled with pre-meiotic germ cells, whereas the testis in M contains elongating spermatids). I imagine these images reflect sterile vs. fertile testes of D50A mutants, but it should be explained more carefully.

**As the reviewer correctly infers, we do see a spectrum/range of phenotype for the D50 mutants (not every individual testis of the same mutant looks exactly the same). Some flies are sterile, some are fertile, so the images reflect the natural variation across the spectrum. We have stated this explicitly in the revised version of the manuscript.**

**Reviewer 2:**

The authors have performed an elegant structure function analysis of the gap junction protein zero population growth (zpg). Prior work from the Tanentzapf lab and other labs has shown that Zpg is expressed in the germline, that zpg mutants have rudimentary gonads, and that Zpg in germ cells interacts with Innexin2 (Inx2) in somatic support cells. The Tanentzapf lab has previously shown that zpg-/- phenotypes could be rescued fully by an exogenous transgene containing the zpg genomic locus inserted at an attB site. This transgene is the workhorse of the current study. The authors make site-directed mutations to the C-terminal domain, the N-terminal domain, phosphorylated Ser and Tyr resides, Cys residues in hemichannel in the extracellular domain, and Asp residues in the channel pore. They then analyzed the ability of these transgenes to rescue germline and somatic defects of the zpg-/- null testes. They find that the C-terminus is required for Zpg to localize to the plasma membrane; that the conserved phosphoresidues are not required for Zpf function; that extracellular Cys residues are involved in membrane localization or protein stability; that Asp mutations produced distinct phenotypes. When the mutation caused a strong phenotype, the effects on GSC maintenance, germline differentiation and somatic differentiation were similar to each other and were similar to the zpf null phenotype. However, weaker "allele" produced intermediate phenotype. Interestingly, D50A and D59A caused defects in spermatid individualization. Overall, the work was of high quality, the data were compelling, all controls were included, the quantification was robust and the work revealed new insights into gap junction function.

1) I have only a couple of comments about the writing. (And on this note, would the authors please provide line numbers and page numbers even if this is not required by the journal because these two things really help the reviewer when writing the critique.)

**Thanks for this suggestion, in the revised manuscript we have added line and page numbers**

2) On p 13 of the PLoS Genet assembled pdf under "Methodology for assessing rescue ability of rescue constructs", this is not a sentence and should be revised: "This reduction meant in zpg mutants rescued with one copy of zpg::GFP GR express Zpg at levels that similar to those seen in heterozygous zpg mutants

(zpg2533/+) where Zpg antibody staining intensity was 34.4% lower compared to that seen in

homozygous wildtype testes (Fig. S2)".

**Thanks for pointing this out, in the revised manuscript we have changed this sentence accordingly.**

3) In the first paragraph of the discussion, the authors write: " Spermatogenesis is particularly suitable for this type of structure function, as encapsulation of the germ cells by somatic cells completely isolates the soma from the environment (Schulz et al., 2002; Fairchild et al., 2015). This means that the soma is fully reliant for its survival on gap junction mediated transport of external cues required for cell differentiation and/or nutrients and metabolites." In my understanding, one face of the somatic cells are exposed to the testis lumen and so in fact are not completed isolated and are not completely dependent on germ cells for nutrients. Can the authors please provide additional support for their statement?

**This is quite an unfortunate mistake on our parts, for which we apologize. We meant to say: “ as encapsulation of the germ cells by somatic cells completely isolates the GERMLINE from the environment (Schulz et al., 2002; Fairchild et al., 2015). This means that the GERMLINE is fully reliant for its survival on gap junction mediated transport of external cues”. We have fixed this in the revision.**

4) Fig. 5 - the circled "tumor" is so small that it is not legible.

**Thanks for pointing this out, we have addressed this issue.**

**Reviewer 3:**

Reviewer #3: In this study, Pesch and colleagues present a structure-function analysis of the germ cell-specific Innexin, Zpg. They use an elegant genomic rescue method to assay an impressive set of mutations in various domains of the protein for function, exploiting the fact that germ cell differentiation is easily observable and provides an excellent readout for gap junction function. Using this approach, they identify several critical domains and residues for Zpg function, as well as novel, late phenotypes that were previously masked by the earlier effects of null mutations.

1) The authors relied extensively on a model of Zpg structure, which was obtained by homology with the only Innexin for which a cryo-EM derived structure exists, from the nematode C Elegans. One issue with this manuscript is that the model is not described in sufficient detail, and the assumptions made in the modelling are not made clear. This makes it hard to judge the extent of confidence in the homology model and how much of the model is speculative. Could the authors provide confidence values for the localization of the specific residues that are predicted to be within the pore domain? Since this model-derived structure is still hypothetical, the authors should use more cautious language in describing the residues’ localization or structural function.

**The reviewer raises an important point. Of note, we have high confidence in the overall folding of Inx4 and the overall location of the residues studied, but we want to point out that the proposed hydrogen bonds and salt bridges are putative, and will need to await further experimental validation. We have highlighted this better in the manuscript. We also provide additional confidence scores of the model, now shown in a new supplemental Figure S1A. This shows that the transmembrane region has high confidence. In addition, we now also added to this supplementary figure a comparison of the homology model with the model obtained through Alphafold, which shows that these models are in excellent agreement (new supplemental Figure S1B and C). As expected, the parts deemed the least reliable from either homology modeling or Alphafold are the parts that diverge between the two structures, but these do not contain the Asp residues that were studied.**

2) A similar caution would be most welcome when describing the tyrosine residues as “conserved phosphorylation sites”. Given that there is no evidence presented that these residues are actually phosphorylated and that mutating them does not result in any germ cell or fertility phenotype, the authors should refer to these as “conserved tyrosine residues”.

**We completely agree with the reviewer and have rephrased to not say “phospho dead” and instead either refer to these mutants by their proper name (Y352F/S356A) or refer to them as “conserved putative phosphorylation sites”**

3) Several mutants show impaired protein localization, which the authors refer to as “cytoplasmic”. Is this indeed cytoplasmic or is the protein within membranes in internal vesicles? None of the mutants should impact on the signal peptide, suggesting that all proteins should be incorporated into the ER during translation. Could the authors address this?

**This is a good question. We have tried to find evidence for incorporation into the ER using an ER marker. The mutant proteins do not appear to be able to exit the ER though. As the figure below illustrates this does appear to be the case, however we are having some technical issues obtaining this data and it’s not currently ready for inclusion in the manuscript.**

**Figure legend: ZPG-GFP (green, 1st and last column) and the ER marker Cnx99A (red, middle and last column) stainings in Wt testes expressing zpg deltaCT::GFP protein. Bottom row magnified picture of upper row. Plot to the left represents the intensity profile obtained from the orange squares in the merge pictures. There is good overlap between ER staining and zpg deltaCT::GFP localization.**

4) Similarly, in the case of the C-terminal deletion (and some others), there is little overlap between the localization of the mutant form of the protein and the endogenous, when both are present. However, the model suggests that Zpg should multimerize, and the residues that are mutated should not impact on this interaction domain which, to this reviewer’s understanding, is within the transmembrane domain. Could the authors speculate and/or exploit their model to predict how the various mutants should impact Zpg interactions with itself? Alternatively, is the defect in trafficking of the mutant proteins, and if so, how does that impact our understanding of the assembly of these multimeric complexes?

**Sorry for not being clearer about this but none of the mutations we made should, as far as we can tell, impact on multimerization. As we state on the manuscript defect in trafficking is one possibility, another is that the mutant proteins make it to the membrane but are not maintained there.**

5) Some of the mutants (Cysteine mutants and C-terminal deletion) partially rescue GSC numbers but no other phenotypes. Can the authors speculate why this would be? Is the localization of these mutants similar to endogenous localization in GSCs which does not appear to be at the membrane/interface with CySCs? This could be assayed by comparing the colocalization specifically in GSCs vs other germ cells. The text mentions that “the zpg cysteine mutant behave indistinguishably from null alleles of zpg”, however the partial GSC number rescues would indicate otherwise.

**This is an interesting question, as we show in figures 3R and 4R there is not statistically significant rescue in the the number of GSCs in the C-terminal deletion or Cysteine mutants, respectively. There is a mild amount of what appears to be rescue in the GFP tagged Cystine mutants (Supplemental Figure 5S) but this difference is very small (about 1 extra GSC per testes) and could easily be due to changes in genetic background. We do agree with the reviewer that saying our mutants are “indistinguishable from null” is perhaps too strong a statement given the data we have. We have rephrased those statements accordingly.**

6) Additionally, the images for the Cysteine mutants consistently show an enlarged hub (as do occasional others). Although this is a known phenotype of germ cell loss (PMID: 8756289), this does not appear to be consistently the case in the zpg mutant alone. Is this simply a reflection of the choice of images, or is there a different effect on hub cell size between the null and Cysteine mutants?

**The reviewer is correct, hubs to appear larger, certainly when compared to wildtype controls but also in comparison to *zpg* null mutants. In the revised version of the manuscript we now specifically mention this in the results section.**

Minor issues:

7) “Vasa is a marker that labels mitotic germ cells, meaning the early stages of spermatogenesis, from germline stem cells (GSCs) to spermatocytes” – spermatocytes are not mitotic.

**Thanks for pointing out this error to us, it has been fixed.**

8) The authors should show an image of DAPI-positive sperm bundles and explain how counts were done, maybe by highlighting what constitutes a sperm bundle on the image.

**We have done as the reviewer suggests by adding the inset in supplemental figure 4F to highlight what constitutes a sperm bundle and adjusting the materials and methods in the sub-section entitled “Image Analysis” in the methods section.**

9) More detail is required for the fertility assays. The methods indicate that the number of offspring were counted but the graph only shows “fertile” or “subfertile”. How were these categories assessed.

**Apologies, we now include this information in the methods section. Specifically we now state that our phenotypic categories mean: Fertile: more than 30 offspring/day; Subfertile: less than 30 offspring/day; Infertile: 0 offspring/day**

10) Cysteine mutants – the Zpg channel is not visible in Figs. 4B-D.

**The reviewer is correct to point this out. We indeed do not detect much Zpg staining in these mutants, except for a few specks that might be background. As we explain in the text in the results section, the poor localisation of these lines drove us to make the GFP tagged version of Cysteine mutants that is presented in Figure E for the analysis of it’s localization, and phenotypically analyzed in supplemental figure Figure S4. In the revised version of the manuscript we specifically mention that “This suggests a possible role for the surface cysteine residues in membrane localization but could also indicate possible issues with protein stability, though, being surface residues, mutations in the surface cysteines are unlikely to impact protein folding or packing.”**

11) “D to A mutations constitute the most significant functional change within our mutagenesis approach” – this is based on the prediction, however the phenotypes do not match this prediction, at least for D50, and arguably also D21. Please amend the text to make clear that this is a prediction, not an observation.

**The reviewer is correct and we fixed the text accordingly.**

12) D59N appears more cytoplasmically-localized, although some is still visible at the membrane. Is this correct or simply the image chosen? The text indicates that D59N localized normally.

**Reviewer is correct to note that there is some cytoplasmic localization, but it is mostly at the membrane. This is directly quantified by our calculation of Pearson co-localization coefficient (Supplemental Figure S8 and results text)**

13) Typo p. 26 “ the soma is fully reliant for its survival on gap junction- mediated transport of external cues”. Did the authors mean the germline?

**Thank you for catching this mistake which was indeed a typo. It has been fixed.**
